# Fecal short-chain fatty acids in non-constipated irritable bowel syndrome: a potential clinically relevant stratification factor based on catabotyping analysis

**DOI:** 10.1080/19490976.2023.2274128

**Published:** 2023-11-01

**Authors:** Giorgio Gargari, Giacomo Mantegazza, Valentina Taverniti, Claudio Gardana, Alice Valenza, Federico Rossignoli, Maria Raffaella Barbaro, Giovanni Marasco, Cesare Cremon, Giovanni Barbara, Simone Guglielmetti

**Affiliations:** a Department of Food, Environmental and Nutritional Sciences (DeFENS), University of Milan, Milan, Italy; b Dipartimento di Scienze Mediche e Chirurgiche, IRCCS Azienda Ospedaliero-Universitaria di Bologna, Bologna, Italy; c Department of Medical and Surgical Sciences, University of Bologna, Bologna, Italy

**Keywords:** Gastrointestinal disorder, abdominal pain, fecal microbiome, diarrhea, fecal type, butyrate, valerate, succinate, metataxonomics, catabotyping

## Abstract

The gut microbiota is believed to be a critical factor in the pathogenesis of IBS, and its metabolic byproducts, such as short-chain fatty acids (SCFAs), are known to influence gut function and host health. Despite this, the precise role of SCFAs in IBS remains a topic of debate. In this study, we examined the bacterial community structure by 16S rRNA gene profiling and SCFA levels by UPLC-MS/MS in fecal samples from healthy controls (HC; *n* = 100) and non-constipated patients (IBS-D and IBS-M; NC-IBS; *n* = 240) enrolled in 19 hospitals in Italy. Our findings suggest a significant difference between the fecal microbiomes of NC-IBS patients and HC subjects, with HC exhibiting higher intra-sample biodiversity. Furthermore, we were able to classify non-constipated patients into two distinct subgroups based on their fecal SCFA levels (fecal catabotype “high” and “low”), each characterized by unique taxonomic bacterial signatures. Our results suggest that the fecal catabotype with higher SCFA levels may represent a distinct clinical phenotype of IBS that could have implications for its diagnosis and treatment. This study provides a new perspective on the intricate relationship between the gut microbiome and bowel symptoms in IBS, underscoring the importance of personalized strategies for its management.

## Introduction

Irritable bowel syndrome (IBS) is a common functional gastrointestinal disorder that affects a large proportion of the population worldwide, with an estimated prevalence of 7–21%, depending on the geographical location and diagnostic criteria used^1^. IBS is characterized by recurrent abdominal pain associated with altered bowel habits, including diarrhea (IBS-D), constipation (IBS-C), or a mixture of both (IBS-M), which can significantly impair the quality of life of affected individuals. Despite its high prevalence and impact, the pathophysiology of IBS still needs to be better understood, and the available treatments result effective only for a limited portion of patients^2,3^.

A growing body of evidence suggests that alterations in the gut microbiota and its metabolic products may play a crucial role in the pathogenesis of IBS^4^. The gut microbiota is a complex community of microorganisms (prevalently bacteria) that colonize the human gastrointestinal tract, interacting with the host immune system, modulating gut motility and secretion, and participating in the digestion and absorption of nutrients^5^. The main metabolic products of the gut microbiota are short-chain fatty acids (SCFAs), a group of organic acids that are mainly produced by the bacterial fermentation of dietary fiber and other carbohydrates in the colon. In particular, the most abundant SCFAs in the human bowel are the bacterial catabolites acetate (C2), propionate (C3), and butyrate (C4), which are reported to reach up to hundreds of millimoles per liter in the proximal colon and cecum^6^. In addition, other organic acids deriving from the bacterial catabolism detected in the human gut are valerate (C5), lactate, and succinate^7^.

SCFAs are essential for maintaining gut health and function since they provide energy to the colonic epithelium, stimulate the production of mucus and antimicrobial peptides, and regulate gut motility and inflammation^8^. Moreover, SCFAs have been shown to have systemic effects beyond the gut, including regulating glucose and lipid metabolism and the modulation of immune function and gut-brain communication^9,10^. Despite the demonstrated beneficial effects of intestinal SCFAs on human physiology and metabolism, the role of these microbial metabolites in IBS remains controversial. Some studies have reported lower levels of SCFAs in IBS patients compared to healthy controls, while others have reported higher levels or no significant differences^11^. This heterogeneity in results may be due to the different subtypes of IBS, as well as the differences in dietary habits, medication use, and other confounding factors that can influence gut microbiota and SCFA production. Recently, we reported chronically elevated SCFA levels in the feces of a small cohort of IBS patients with diarrhea (*n* = 11; IBS-D) compared to IBS patients with constipation (*n* = 12; IBS-C) and healthy controls (*n* = 25) over a period of 16 weeks^12^. Similarly, a meta-analysis by Sun et al. evidenced a significant increase of fecal butyrate in IBS-D patients compared to healthy controls^13^. Nonetheless, contradicting results are also reported, such as reduced SCFAs^14^, and improved gastrointestinal symptoms upon oral intake of butyrate in IBS-D patients^15,16^.

To investigate the role of the intestinal microbiome in patients with IBS, we studied fecal short-chain fatty acid (SCFA) levels and bacterial community structure in a cohort of 240 non-constipated patients (IBS-D and IBS-M; NC-IBS) recruited from 19 hospitals in Italy in comparison to healthy control (HC) subjects. Our findings suggest that NC-IBS patients can be stratified into distinct subgroups based on fecal SCFA levels (catabotypes), which are characterized by specific taxonomical bacterial signatures and clinical profiles.

## Results

### The fecal microbiome of NC-IBS patients and HC subjects differs significantly

**Figure 1.**
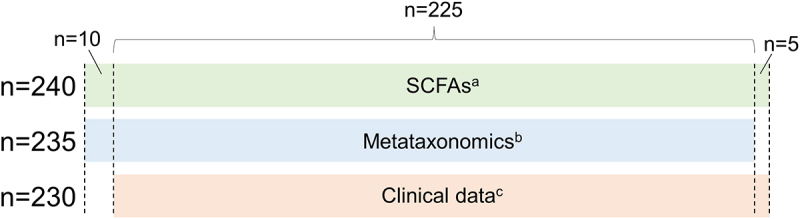
Diagram reporting the number of patients for which specific indicated data are available. (a), quantification of organic acids in feces; (b), metataxonomics of fecal samples through 16S rRNA gene profiling; (c), abdominal pain and bowel habits data.

**Figure 2.**
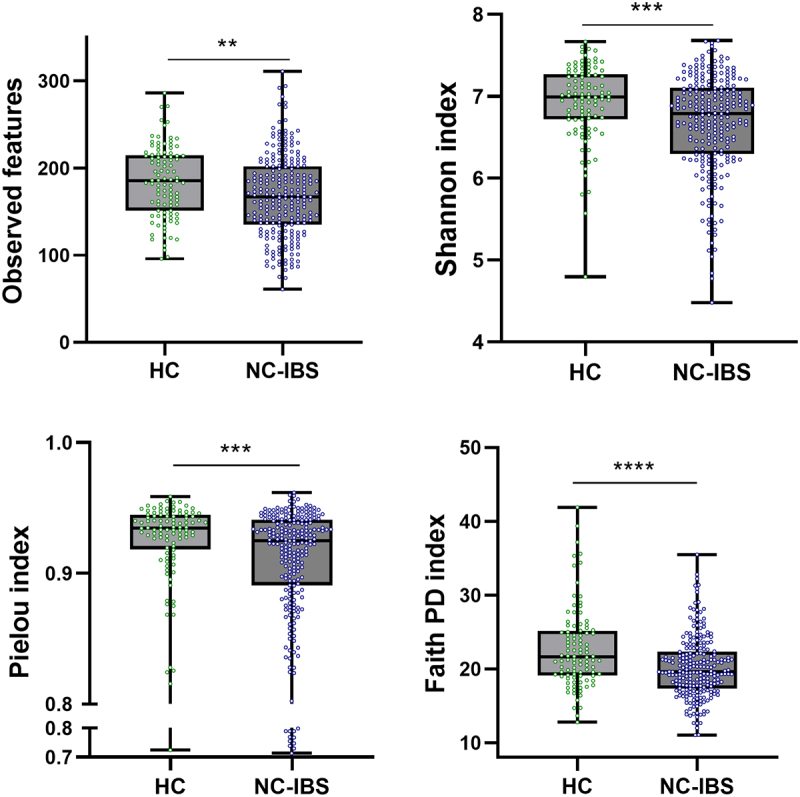
Comparison of the intra-sample biodiversity of the fecal bacterial communities between non-constipated patients (NC-IBS; *n* = 235) and healthy control subjects (HC; *n* = 100) according to four different α-diversity indexes. Statistics is according to the Mann-Whitney test. **, *P* < .01; ****, *P* < .0001).

**Figure 3.**
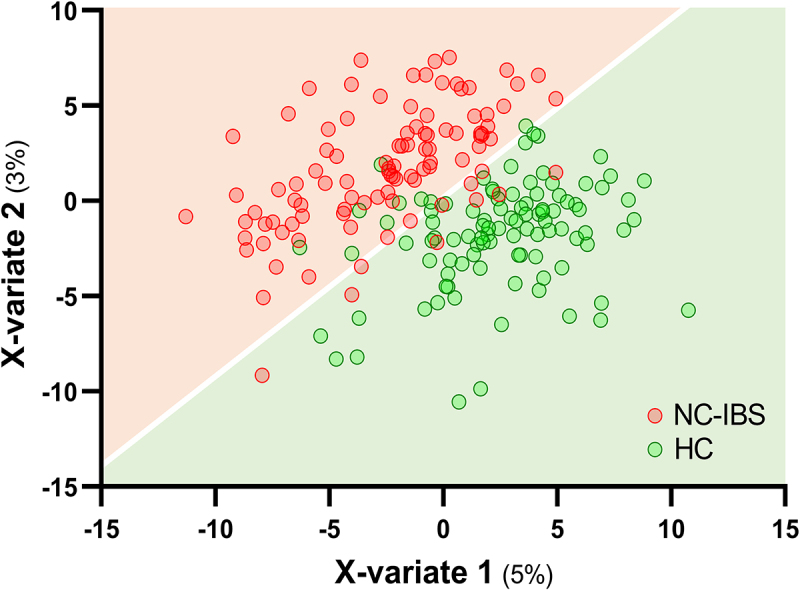
PLS discriminant analysis (PLSDA) with prediction background for non-constipated IBS patients (NC-IBS) and healthy control subjects (HC). The Receiver Operating Characteristic (ROC) curve of the PLSDA model is shown in supplementary Figure S1.

Metataxonomic analysis evidenced several significant differences between HC (*n* = 100) and NC-IBS patients (*n* = 235; Figure 1). Intra-sample biodiversity (α-diversity) was found to be significantly higher in HC as measured with four different indexes: observed-features (measure of richness), Shannon’s entropy (considering both evenness and richness), Pielou’s (measure of evenness), and Faith’s phylogenetic diversity (PD) (Figure 2). Conversely, we did not find significant differences in β-diversity between IBS and HC as determined through both unweighted and weighted UniFrac (Not shown). Nonetheless, it was possible to separate NC-IBS from HC samples by performing a PLS discriminant analysis (PLSDA) based on the CLR-transformed abundance of bacterial taxa in fecal samples (outcome HC vs. NC-IBS: 0.96; accuracy: 0.67; Figure 3; Supplementary Figure S1).

The analysis of metataxonomic data was also conducted while keeping the IBS-D and IBS-M patients separate. The results revealed no significant differences between these two subgroups, both in alpha-diversity and taxonomic community structure (see Supplementary Figure S2), thus supporting the decision to analyze these two subgroups together.

**Figure 4.**
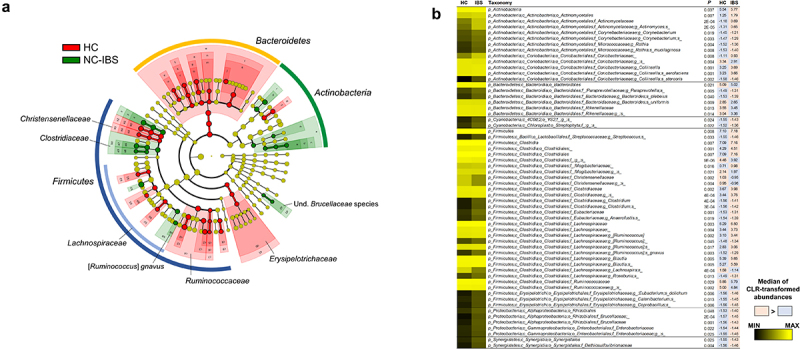
Bacterial taxa in fecal samples exhibiting a significantly different abundance between individuals with non-constipated irritable bowel syndrome (NC-IBS) and healthy subjects (HC). (a) cladogram based on LEfSe analysis (LDA scores for the single taxa are reported in Supplementary Figure S3). (b) significantly different taxa determined through the Mann-Whitney test carried out with CLR-transformed bacterial abundances. Higher and lower abundances for each taxon are reported with a red and cyan background, respectively. The black and yellow heatmap represents the mean CLR-transformed abundances of the reported taxonomic units. The taxonomic lineage of each taxon is shown: p, phylum; c, class; o, order; f, family; g, genus; s, species.

Several taxa were found to be differently represented between NC-IBS and HC samples. Specifically, the phylum Bacteroidetes, the Firmicutes families Ruminococcaceae and Christensenellaceae, and the order Erysipelotrichales were underrepresented in NC-IBS samples, as revealed by LEfSe analysis (Figure 4a; Supplementary Figure S3). Conversely, we observed an overrepresentation of the Actinobacteria genera *Rothia* and *Collinsella* (in particular, the species *C. aerofaciens*), and several Firmicutes taxa, including the genera *Clostridium* (family Clostridaceae) and [*Ruminococcus*] (now reclassified as *Mediterraneibacter*; family Lachnospiraceae), and the species [*Ruminococcus*] *gnavus* in NC-IBS samples (Figure 4a; Supplementary Figure S3).

Due to the compositional nature of 16S rRNA gene profiling data [17], we inferred the bacterial taxa that were differently represented between NC-IBS and HC samples after CLR-transformation of taxonomic abundances. The obtained results confirmed several findings obtained with the LEfSe analysis, including the overrepresentation of the species *C. aerofaciens* and [*R*.] *gnavus* in NC-IBS samples (Figure 4b). Overall, at the higher taxonomic levels, the analysis of CLR-transformed data showed that NC-IBS patients had increased Actinobacteria and Firmicutes (order Clostridiales), and decreased Bacteroidales compared to HC. At the family level, the main bacteria overrepresented in the IBS patients were Actinomycetaceae, Clostridiaceae, Lachnospiraceae, Brucellaceae, and Enterobacteriaceae. Additionally, within Bacteroidetes, we found that the species *Bacteroides uniformis* was more abundant in HC, whereas *Bacteroides plebeius* was more abundant in NC-IBS (Figure 4b).

**Figure 5.**
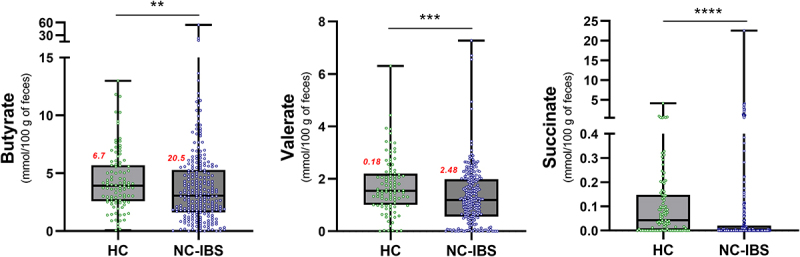
Organic acids in fecal samples exhibiting a significantly different abundance between individuals with non-constipated irritable bowel syndrome (NC-IBS) and healthy subjects (HC). The statistical analysis was performed using the Mann-Whitney test and indicated a statistically significant difference compared to HC samples. **, *P* < .01; ***, *P* < .001; ****, *P* < .0001. Red numbers refer to the σ^2^ variance of the corresponding data.

Subsequently, we quantified the concentration of the primary organic acids derived from bacterial catabolism in the human gut, including acetate, butyrate, propionate, valerate, lactate, and succinate, in fecal samples collected from 100 HC and 240 NC-IBS individuals (Figure 1). Acetate and butyrate were the most abundant organic acids detected in most samples, with propionate being the most abundant in ten NC-IBS patients and one HC, and succinate being the most abundant in four NC-IBS patients and one HC. Valerate and lactate were the most abundant organic acids in four and one NC-IBS patients, respectively. NC-IBS patients had a significantly lower abundance of butyrate (median concentrations of 3.9 vs. 3.1 mmol/100 g of feces; *P* = 0.007 according to the Mann-Whitney test), succinate (0.04 vs. 0.00 mmol/100 g of feces; *P* = 3.8 × 10^–7^), and valerate (1.5 vs. 1.2 mmol/100 g of feces; *P* = 4.3 × 10^–4^) compared to HC subjects (Table 1; Figure 5). The propionate/butyrate ratio, which was proposed as a potential biomarker for IBS^17,18^, was not significantly different between IBS and HC samples. Furthermore, we found no significant differences in fecal organic acids between IBS-D and IBS-M subtypes (Supplementary Figure S2).

**Table 1.** Concentration of organic acids in fecal samples collected from healthy subjects (HC) and non-constipated patients (NC-IBS). Data for NC-IBS samples are also shown after stratification in catabotypes FC-H and FC-L. All data are reported in mmol/g of feces. Prp/but, ratio between the concentrations of propionate and butyrate. Statistics is according to Mann-Whitney test and indicate statistically significant difference compared to HC samples. *, *P* < .05; **, *P* < .01; ***, *P* < .001; ****, *P* < .0001.

	HC (*n* = 100)		NC-IBS (*n* = 240)			FC-H (*n* = 128)			FC-L (*n* = 112)		
	Median	*Mean*	Median	*Mean*		Median	*Mean*		Median	*Mean*	
Acetate	3.21	*4.19*	2.95	*3.44*		3.97	*4.52* **		2.07	*2.21* ****	
Butyrate	3.92	*4.35*	3.07	*3.96* **		4.97	*5.99* ***		1.57	*1.64* ****	
Propionate	1.21	*1.46*	1.32	*1.46*		1.71	*2.09* ****		0.7	*0.74* ****	
Valerate	1.54	*1.71*	1.19	*1.33* ***		1.86	*1.87*		0.65	*0.72* ****	
Lactate	-	*0.03*	-	*0.09*		-	*0.16*		-	*0.02* ****	
Succinate	0.04	*0.14*	-	*0.28* ****		-	*0.24* ****		-	*0.33* ****	
Prp/But	0.34	*0.41*	0.41	*0.83*		0.38	*0.93*		0.48	*0.73* *	

Overall, our results indicate that NC-IBS patients exhibit significant alterations in their fecal microbiome compared to healthy individuals. Specifically, our data suggest a general expansion of Actinobacteria, particularly the species *C. aerofaciens*, as well as a reduction in Bacteroidetes, butyrate, valerate, and succinate in the fecal microbiome of NC-IBS patients.

### Stratification based on the fecal levels of SCFAs revealed two distinct microbiome clusters in NC-IBS

**Figure 6.**
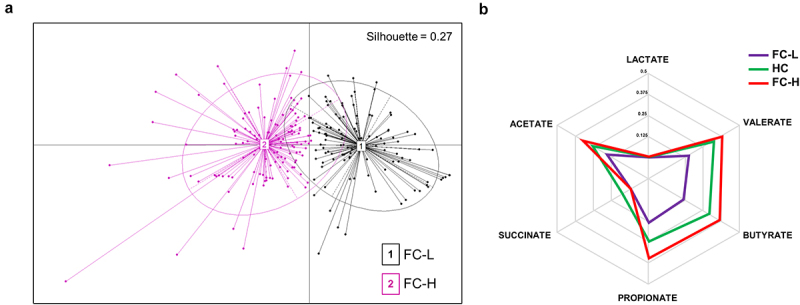
Stratification analysis of non-constipated IBS patients (NC-IBS) based on the fecal levels of the organic acids. (a) principal-coordinate analysis (PCoA) generated through catabotyping based on the fecal concentrations of acetate, butyrate, propionate, lactate, succinate, and valerate. (b) spider chart based on the abundances of bacterial catabolites expressed as relative abundance.

We performed an unsupervised clustering analysis of the 240 NC-IBS patients under investigation using principal coordinate analysis (PCoA) based on the concentrations of six microbial catabolites in their fecal samples: acetate, butyrate, propionate, lactate, succinate, and valerate (catabotyping analysis). Based on the highest Silhouette coefficient (SI = 0.27) and variance ratio criterion (CH index = 77), we were able to classify the IBS samples into two distinct clusters, which we named fecal catabotype “high” (FC-H; *n* = 128) and “low” (FC-L; *n* = 112) (Figure 6a). The FC-H group was characterized by significantly higher concentrations of all six metabolites, except for succinate, compared to FC-L. Additionally, the fecal levels of acetate, butyrate, and propionate were lower in HC than in FC-H, while succinate was higher. Notably, HC subjects had significantly higher fecal concentrations of all metabolites compared to FC-L NC-IBS patients (Table 1). Moreover, we observed that the propionate/butyrate ratio was higher in FC-L (median value of 0.48) compared to HC (0.34; *P* = 0.011) and FC-H (0.38; *P* = 0.066). These findings suggest that NC-IBS patients can be categorized into two clusters characterized by higher and lower fecal SCFA levels than HC, as evidenced by the spider chart based on the relative abundances of bacterial catabolites (Figure 6b). Furthermore, the identified metabotypes do not overlap with IBS subtypes, as it emerged that IBS-D samples distributed in 60% and 40% between FC-H and FC-L, respectively, while IBS-M samples distributed in 45% and 55% between FC-H and FC-L, respectively.

**Figure 7.**
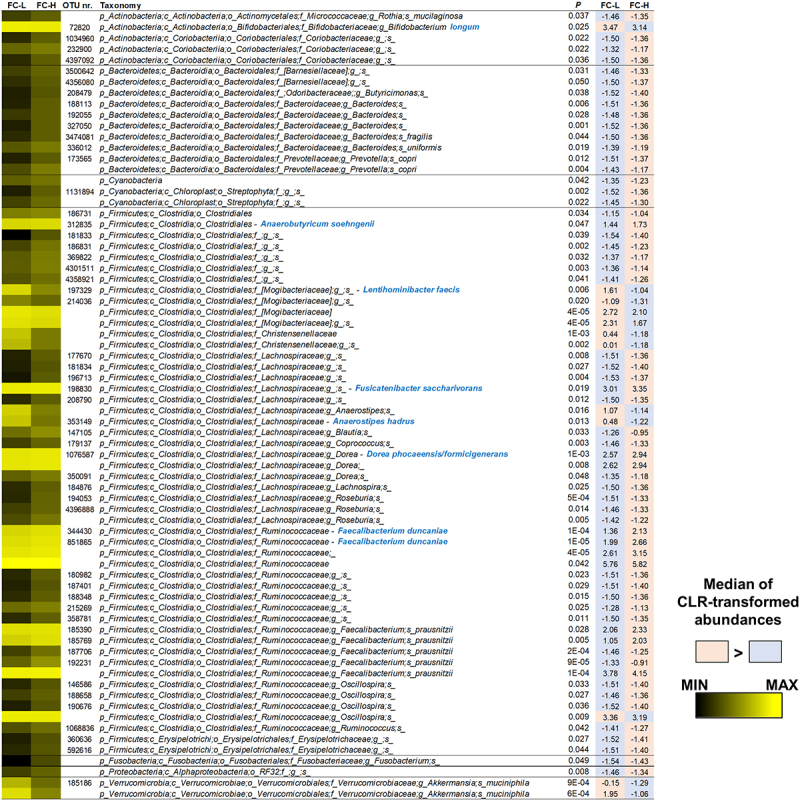
Bacterial taxa in fecal samples of NC-IBS patients exhibiting a significantly different abundance between catabotypes FC-H and FC-L. Significantly different taxa have been determined through the Mann-Whitney test carried out with CLR-transformed bacterial abundances. The black-yellow heatmap represents the mean CLR-transformed abundances of the reported taxonomic units. Higher and lower abundances for each taxon are reported with a red and cyan background, respectively. The taxonomic lineage of each taxon is shown: p, phylum; c, class; o, order; f, family; g, genus; s, species. Taxonomic names written in blue were determined through a manual BLASTN search in GenBank using the sequence of the corresponding reads.

Since intestinal SCFA levels directly result from microbial metabolism in the gut, we compared the fecal bacterial community structure of the IBS subgroups FC-H and FC-L. Statistical analysis of CLR-transformed abundances revealed 73 bacterial taxa with different representations, including 54 OTUs (Figure 7). Only 12 taxa were more abundant in FC-L than FC-H, including OTUs ascribed to the species *Bifidobacterium longum* (phylum Actinobacteria), *Lentihominibacter faecis*, *Anaerostipes hadrus*, and an undefined *Oscillospira* species (order Clostridiales), and *Akkermansia muciniphila* (Figure 7). Conversely, among the most abundant taxa overrepresented in FC-H, we found several bacteria known to ferment fiber to produce SCFAs, such as *Anaerobutyricum soehngenii*, *Fusicatenibacter saccharivorans*, *Dorea phocaeensis*/*formicigenerans*, *Faecalibacterium duncaniae*, and *Faecalibacterium prausnitzii* (Figure 7).

Comparison with HC showed that NC-IBS catabotypes had lower α-diversity (Supplementary Figure S4). In addition, we found that 102 bacterial taxa (including 62 OTUs) significantly distinguished HC from FC-L samples. In contrast, the number of differently represented bacterial taxa between HC and FC-H was 276 (including 206 OTUs) (Supplementary Figure S5). In general, most of the significantly different bacterial taxa were overrepresented in the IBS catabotypes compared to HC samples (86 and 263 significantly different taxa respectively increased in FC-L and FC-H compared to HC). Specifically, both FC-L and FC-H had significantly more Firmicutes than HC, mostly due to the overrepresentation of the order Clostridiales and the family Lachnospiraceae within it. Also, Actinobacteria (particularly the species *Collinsella aerofaciens*) showed higher abundance in both IBS bacterial catabotypes than HC. On the contrary, dominant members of the phylum Bacteroidetes were depleted in the IBS groups compared to HC, such as the order Bacteroidales and the species *Bacteroides uniformis* reduced in FC-L, and the family Rikenellaceae depleted in FC-H. Notably, the butyrate producer *F. prausnitzii* was higher in FC-H and lower in FC-L compared to HC (Supplementary Figure S5).

Overall, these data show that IBS patients can be stratified according to the fecal level of SCFAs into two groups (catabotypes), which the diverse representation of several bacterial taxa can distinguish. In particular, the catabotype FC-H, which had significantly higher concentrations of SCFAs, was characterized by the overrepresentation of many taxonomic units (including numerous fiber degraders and SCFA producers) compared to both HC and the NC-IBS group FC-L.

### Fecal catabotypes may be clinically relevant

Subsequently, we compared the IBS subgroups FC-H and FC-L in relation to the clinical parameters registered two weeks before fecal collection. We considered abdominal pain (Numeric Pain Rating Scale, NRS scale), fecal type, and stool frequency. Notably, patients in the catabotype FC-H (*n* = 120) had higher levels of abdominal pain (normal distribution: mean value of 3.8 vs. 3.2; *P* < .01) and fecal type (non-normal distribution: median value of 4.9 vs. 4.4; *P* < .0001) than those in the FH-L catabotype (*n* = 99) (Table 2a).

**Table 2.** Clinical symptoms in the non-constipated IBS patients in the PROBE-IBS/2 cohort of the fecal catabotypes FC-L and FC-H. (a) comparison of the FC-H and FC-L catabotypes at baseline. Statistics is according to the Mann-Whitney U test (fecal type and evacuation number) or the unpaired t-test (abdominal pain), depending on the normal distribution of data. (b) comparison between the initial (baseline) and final (after 16 weeks) visit. Statistics is according to the Wilcoxon signed-rank test (fecal type and evacuation number) or the unpaired t-test (abdominal pain) depending on the normal distribution of data. *, *P* < .05; **, *P* < .01.

		FC-H (*n* = 125)			FC-L (*n* = 105)					
A		Median		*Mean*	Median	*Mean*				
Abdominal pain (NRS)		3.9		*3.8*	2.9	*3.2* **				
Fecal type		4.9		*4.8*	4.4	*4.3* ****				
Evacuation number		1.8		*1.9*	1.6	*1.9*				
			Median				*Mean*			
B			baseline		week 16		*baseline*		*week 16*	
FC-H (*n* = 114)	Abdominal pain (NRS)		3.7		2.8		*3.7*		*3.2* **	
	Fecal type		4.9		4.5		*4.9*		*4.6* **	
	Evacuation number		1.8		1.6		*1.9*		*1.9*	
FC-L (*n* = 88)	Abdominal pain (NRS)		2.9		2.4		*3.1*		*2.8*	
	Fecal type		4.4		4.2		*4.3*		*4.1*	
	Evacuation number		1.6		1.6		*1.9*		*1.9* *	

**Figure 8.**
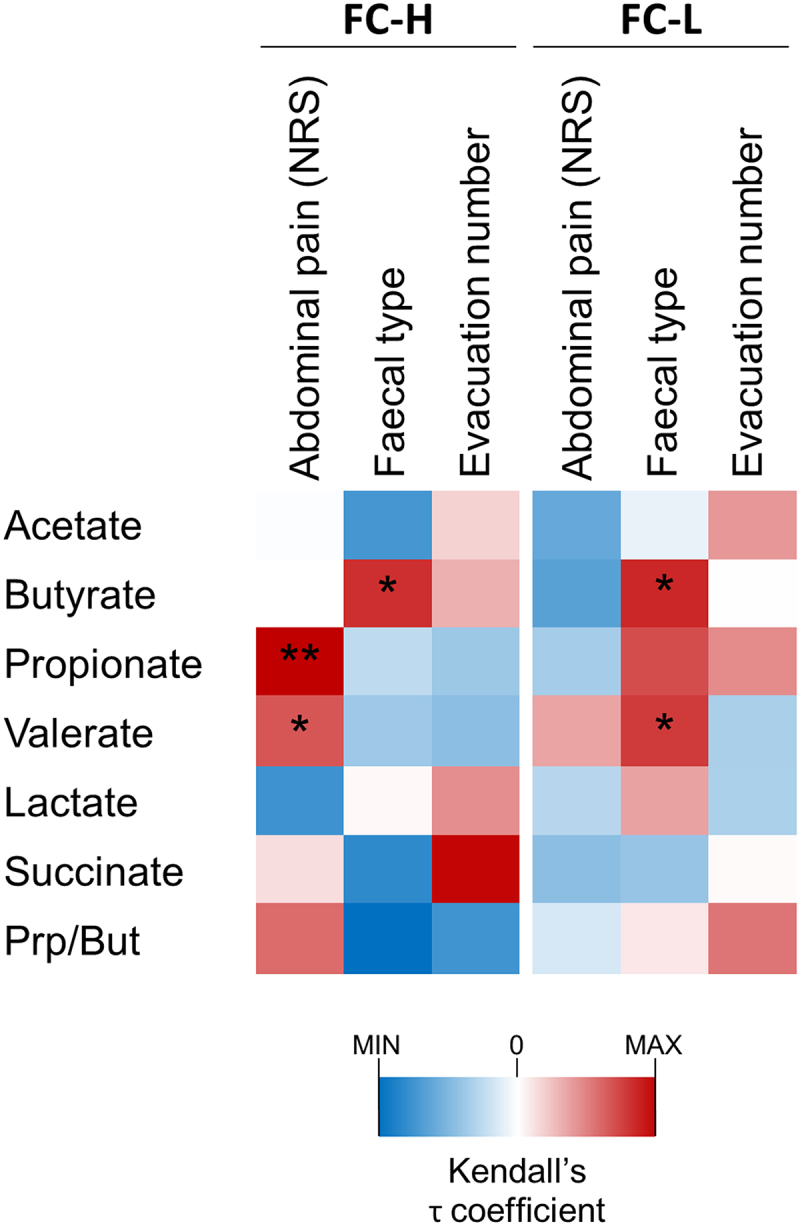
Correlation analysis between fecal organic acids and IBS symptoms in catabotypes FC-L (*n* = 105) and FC-H (*n* = 125). The heatmap represents the τ coefficient of Kendall rank correlation. Asterisks indicate significance in the Kendall rank correlation: *, *P* < .05; **, *P* < .01. NRS, numeric rating scale.

Subsequently, we performed a Kendall’s correlation analysis, which showed a significant positive association of abdominal pain toward valerate and propionate, and succinate toward evacuation number, but only in the FC-H catabotype (Figure 8). In addition, fecal type and butyrate correlated positively in both catabotypes, whereas fecal type and valerate correlated only in the catabotype FC-L (Figure 8).

**Figure 9.**
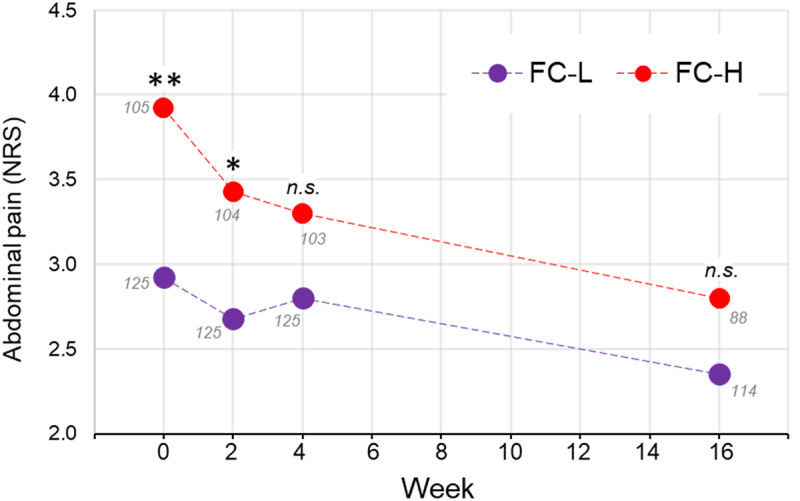
Progression of abdominal pain over 16 weeks in the PROBE-IBS/2 cohort of non-constipated IBS patients stratified in the fecal catabotype FC-L and FC-H. Each point refers to the median value of the numeric rating scale (NRS) for pain assessment. Statistics is according to the Mann-Whitney test between FC-L and FC-H at each specific time point. The numbers in gray close to each point refer to the number of patients. **, *P* < .01; *, *P* < .05; *n.s*., not significant.

Finally, we analyzed the clinical data of the PROBE-IBS/2 cohort of IBS patients for 16 weeks during which they followed a conventional therapeutic protocol for IBS based on dietary and lifestyle recommendations (*n* = 202). Interestingly, the patients in the FC-H group responded better than those in FC-L. In fact, over a period of 16 weeks, FC-H patients reported a significant reduction of abdominal pain and fecal type, whereas only the stool frequency decreased in the FC-L group (Table 2b). In particular, the initial difference between FC-L and FC-H in abdominal pain was not anymore significant after 16 weeks due to a better responsiveness of the FC-H patients during the first 4 weeks (Figure 9).

Overall, these data showed that the fecal catabotypes FC-H and FC-L are characterized by different IBS symptom severity and suggest that the direct association between fecal SCFA and IBS symptoms can be stronger in the FC-H catabotype.

## Discussion

Numerous studies investigated the role of the intestinal microbial ecosystem in IBS, providing several different hypotheses on the potential microbial signatures for this condition^19^. The fact that IBS is a remarkably heterogeneous condition both at the etiological level and in its symptomatic manifestations plausibly justifies the contradicting findings and suggests the need to distinguish between potentially different subtypes within IBS. In this context, bowel habits represent the most commonly used criterion to categorize among IBS patients. In support of this discrimination strategy, several studies highlighted significant differences between IBS patients with diarrhea compared to those with constipation, which seem to be particularly clear at the level of the intestinal microbiome in terms of both specific taxa^20^ and microbial metabolites such as SCFAs^21^. Therefore, in order to reduce the existing variability in the context of IBS, in this study, we restricted our analysis to IBS patients with diarrhea (IBS-D and IBS-M), excluding constipation-predominant IBS. Two factors substantiate the inclusion of mixed-type IBS within a group with IBS-D: (i) existing scientific literature demonstrating similarities in characteristics and responses to clinical treatments among patients with IBS lacking constipation^22,23^; (ii) the absence of significant differences in the taxonomic community structure of the fecal microbiome and fecal levels of SCFAs between IBS-D and IBS-M, as revealed in this study.

The comparison of the fecal bacterial communities of a cohort of 235 NC-IBS patients with 100 age- and sex-matched healthy controls highlighted some significant differences that already emerged in the literature, such as a significantly lower alpha-diversity^20^ and an expansion of the Actinobacteria phylum^24,25^. Furthermore, here we observed the increase in Firmicutes (particularly the order Clostridiales) and the depletion of Bacteroidetes (particularly the order Bacteroidales) in NC-IBS compared to HC, as reported in IBS also by two systematic reviews^26,27^, supporting the notion that these taxa may represent valid microbial signatures in the fecal microbiota of patients with IBS.

Available research data suggest that the primary bacterial metabolites of the human gut, i.e., the SCFAs, may play a crucial role IBS in pathogenesis. In particular, several mechanistic studies suggest the causal involvement of intestinal SCFAs in the pathophysiology of diarrhea-predominant IBS as a result of the direct effect of these organic acids on serotonin synthesis, colon motility, and visceral hypersensitivity^14,28–31^. As a result, it has been suggested that regulating colonic SCFA levels could serve as a potential therapeutic target for IBS^11,17,32^. With this in mind, we quantified the main bacterial catabolites in the feces of the NC-IBS patients. The analysis of the obtained data revealed a lower concentration of butyrate, succinate, and valerate in their fecal samples compared to the HC subjects. Elevated succinate levels in the gut were associated with inflammation^33^, and were reported in IBS-D patients with symptom exacerbation^34^ and IBS-D compared to IBS-C^35;^ nonetheless little is known about succinate concentration in IBS compared to the healthy population. Valerate, shown to reduce autoimmunity by enhancing IL-10 and suppressing Th17 cells^36^, was reported to be positively associated with fecal type and significantly higher in patients with IBS-D than in those with IBS-C^12,13^. Fecal valerate levels were also shown to be correlated with visceral sensation (abdominal pain or visceral hypersensitivity) in IBS-D patients, but a significant difference compared to healthy control subjects was not found^17,37^. A much broader, nonetheless partly contradicting, literature is available for butyrate. As opposite to the data presented here, in a previous publication, we reported that fecal butyrate levels, calculated as the median values of five measurements conducted over 16 weeks, were significantly higher in a smaller group of IBS-D patients (*n* = 11) than in IBS-C subjects (*n* = 12) and healthy controls(*n* = 23)^12^. Also in the meta-analysis by Sun et al., fecal butyrate was shown higher in patients with IBS-D than in HCs^13^. Nonetheless, in other studies, fecal butyrate levels in IBS-D were reported to be not dissimilar from HC^18,38^.

The available literature suggests that the subtype, particularly IBS-D versus IBS-C, may partly explain the ambiguous results regarding the differences in colonic levels of butyrate and other SCFAs between IBS patients and the healthy population. However, our findings demonstrate a wider variability in fecal SCFA levels in NC-IBS compared to HC (for instance, a σ^2^ variance of 20.5 in NC-IBS versus 6.7 in HC samples for butyrate; Figure 5). This let us speculate about the possibility of different subgroups within the NC-IBS patient population, for which SCFAs may have variable clinical relevance. To test this hypothesis, we analyzed the data of fecal SCFA levels with an algorithm already adopted for the unsupervised stratification of human individuals based on bacterial metataxonomic data of their fecal microbiota, known as “enterotyping”^39^. The enterotype concept is a controversial theory^40^ suggesting that the gut microbiome can be classified into a small number of distinct community types based on the abundance of specific bacterial groups. These enterotypes have been proposed to be relatively stable and to play a role in the host’s health and disease risk, therefore potentially relevant in clinical practice^41^. In analogy with the enterotype concept, here we aimed to assess the hypothesis that IBS patients could be stratified in catabotypes, i.e., distinct groups of subjects based on the fecal levels of the main organic acid produced by the microbial catabolism in the human colon. The application of catabotyping permitted the stratification of the 240 IBS patients here investigated into two groups with sharply different levels of fecal SCFAs, which for one group were significantly higher (catabotype FC-H) and for the other (catabotype FC-L) significantly lower than those in HC subjects. Notably, we found that the catabotype FC-H showed higher levels of abdominal pain and fecal type compared to FC-L suggesting that an association between IBS symptoms and catabotypes may exist. Nonetheless, we cannot conclusively state the direction of the observed association between SCFAs and IBS symptoms since it can either be possible that alterations in gut motility and sensitivity lead to changes in SCFA production or vice versa. According to available literature, discrepancies are evident regarding the possible association between SCFAs and bowel symptoms. Ringel-Kulka et al. reported that total fecal SCFA levels correlated negatively with colon transit time and positively with stool frequency^18^. According to Tana et al., high acetate and propionate levels are associated with significantly worse abdominal symptoms^42^. Nonetheless, Wang et al. reported that SCFA signatures were not consistently associated with IBS severity over time^43^. Furthermore, administering SCFAs in the ileum increased ileal motility and abdominal pain in humans^44^ and promoted visceral hypersensitivity in rats^45,46^. However, in the distal colon, butyrate was shown to increase and propionate to decrease the rate of colonic propulsion in guinea pigs^47^. Exogenous administration of butyrate was also shown to increase colon motility in a mouse model of IBS^28^. Nonetheless, recently, a prospective multicenter clinical trial involving 3000 non-hospitalized IBS patients showed that the oral administration of sodium butyrate may effectively relieve the IBS symptoms^16^.

In this study, we demonstrated that the fecal microbiota of individuals with the FC-H catabotype, who have a greater abundance of SCFAs in their feces, differed significantly (more than FC-L) from those of HC, precisely due to the overrepresentation of multiple bacterial taxa. This finding suggests that the FC-H catabotype may represent a subset of IBS patients with a higher degree of dysbiosis. Recently, Vervier et al. used shotgun metagenomics to analyze fecal samples from 56 IBS-D and IBS-M patients before and after a low-FODMAP diet. Through unsupervised clustering, they identified two fecal microbiota subtypes with different clinical responses to the dietary therapy. In particular, they reported a significantly enhanced responsiveness in the “pathogenic-like” subtype (IBS^P^), enriched in Firmicutes and genes for carbohydrate metabolism^23^. Our data suggest that the FC-H catabotype may share similarities with the IBS^P^ subtype proposed by Vervier et al. Therefore, we hypothesize that the FC-H catabotype may represent a subgroup of NC-IBS patients who could benefit the most from a low-FODMAP diet or other dietary interventions targeting the fiber-fermenting microbial components of the gut microbiota. In substantiation of this hypothesis, our study findings indicate that adherence to the dietary guidelines provided to patients in the PROBE-IBS/2 trial, which included the avoidance of fibrous, large-leaf vegetables, legumes, fruits with high fiber content, and whole grains (further information on the recommendations on lifestyle and dietary habits are available in the Supplementary Material file), yielded a more substantial amelioration in abdominal pain and fecal consistency within the FC-H subgroup. Contextually, Eetemadi and Tagkopoulos^48^ analyzed metataxonomic data from six different low-FODMAP intervention trials and concluded that IBS patients who are likely to respond better to a low-FODMAP diet are characterized by a higher abundance of putative genes related to SCFA metabolism pathways^48^. Likewise, Chumpitazi and colleagues documented that in children with IBS who responded positively to the FODMAP diet, there were elevated levels of particular microbial groups, such as Ruminococcaceae and *Faecalibacterium prausnitzii*, which have been linked to higher rates of saccharolytic metabolism^49^. Furthermore, our correlation analysis revealed a significant positive association between butyrate and fecal type, and between propionate and valerate levels and abdominal pain exclusively in the FC-H catabotype. This observation supports the hypothesis that only in patients with higher concentration, fecal SCFAs may play a role in IBS pathogenesis and could represent a valid target to enhance diagnosis and guide toward potentially more effective treatment options. Specifically, we speculate that higher levels of fecal organic acids in NC-IBS patients may indicate a greater likelihood of success for therapeutic interventions targeting the microbiome. In addition to the low-FODMAP diet, which has significant limitations in long-term sustainability, this subset of IBS patients may represent the population most likely to derive substantial benefits from treatments such as antibiotics (e.g., as rifaximin), interventions involving probiotics or live biotherapeutics (e.g., *Blautia hydrogenotrophica*,^50^, and fecal microbiota transplantation (FMT), the efficacy of which in IBS is controversial^51^. Concerning FMT, we can postulate that in IBS patients where the colonic microbial ecosystem is a major contributor to symptoms (as we hypothesized here, particularly in FC-H patients), FMT may offer the most significant and potentially enduring advantages.

In conclusion, this study investigated the role of SCFAs in IBS, and specifically explored the presence of a subgroup of non-constipated patients characterized by higher levels of SCFAs (that we named catabotype FC-H). We hypothesized that the FC-H subgroup may represent a distinct clinical phenotype of IBS with specific clinical features. In order to understand if the identification of this catabotype may have implications for the diagnosis and treatment of IBS, further research efforts should be first directed toward the development of a standardized analytical protocol and the identification of a threshold for fecal organic acids that can help establish whether a non-constipated IBS patient falls into this category of patients.

Overall, our study offers novel perspectives on the intricate relationship between the gut microbiome and bowel symptoms in IBS, emphasizing the importance of tailored strategies for its treatment.

## Patients and methods

### Study participants and clinical assessment

The present study included a total of 240 patients diagnosed with non-constipated [i.e., IBS with predominant diarrhea (IBS-D) or IBS with mixed bowel habits (IBS-M); NC-IBS] according to Rome IV criteria, recruited in 19 Italian hospitals. These subjects were from the cohort of the PROBE-IBS/2 trial (ClinicalTrials.gov Identifier NCT03449628). In addition, we included 100 healthy controls matching the IBS patients of the PROBE-IBS/2 cohort in terms of age and sex. The characteristics of the study populations are summarized in Table 3. The standard 11-point numeric rating scale (ranging from 0, indicating no pain, to 10, indicating the worst possible pain) was used to measure abdominal pain in IBS patients at two-week intervals. Finally, we assessed stool frequency and form using the Bristol stool chart. The patients’ abdominal pain and bowel habits were monitored over 16 weeks. At recruitment, patients received the recommendations on life-style and eating habits described in the supplementary material file (Supplementary Methods).

**Table 3.** Demography and clinical information of subjects involved in the study. NC-IBS, cohort of non-constipated irritable bowel syndrome patients of the PROBE-IBS/2 trial. HC, healthy control subjects.

	NC-IBS		HC
	baseline	week 16	
Number of patients	240	202	100
Age (years; mean ± std. dev.)	36 ± 12	35 ± 11	39 ± 13
Female sex [n (percentage)]	148 (62%)	123 (61%)	54 (54%)
Diagnosis criteria (Rome)	IV	IV	/
Irritable bowel syndrome type			
IBS-D [n (percentage)]	136 (57%)	121 (60%)	/
IBS-M [n (percentage)]	104 (43%)	81 (40%)	/

### Ethics statement

The ethical approvals were obtained for trial PROBE-IBS/2 by the Committee of the coordinating center (Hospital S. Orsola Malpighi – Bologna - Approval identification number 67/2017/U/Sper on June 13, 2017) and by the Ethics Committee of each participating site. The trial was carried out in compliance with the Declaration of Helsinki and the principles of good clinical practice. A written informed consent was provided by all participants.

### Organic acid quantification in fecal samples

We quantified organic acids in fecal samples obtained from 240 IBS patients of the PROBE-IBS/2 cohort at recruitment and from 100 healthy controls. The organic acids measured included acetate, butyrate, propionate, valerate, lactate, and succinate. Ultra-Performance Liquid Chromatography – High-Resolution Mass Spectrometry (UPLC-HR-MS) was used to detect and quantify these molecules in fecal samples. This technique utilized an Acquity UPLC separation module (Waters, Milford, MA) coupled with an Exactive Orbitrap MS and a HESI-II probe for electrospray ionization (Thermo Scientific, San Jose, CA), as previously described^12^.

### Metataxonomics of fecal samples

Metataxonomic analysis was performed on fecal samples collected from all 235 patients of the PROBE-IBS/2 cohort and 100 control samples from healthy subjects by profiling the 16S rRNA gene. Fecal samples were collected by the patients and delivered to the reference hospital within 24 h with instructions to store them in a refrigerator (without freezing) before delivery. Subsequently, the samples were promptly frozen at −80°C and ultimately transferred in dry ice to the centralized laboratory at the University of Milan for analysis. Total DNA was extracted from 150 mg of feces using the QIAsymphony PowerFecal Pro DNA Kit (Qiagen, Milan, Italy) in accordance with the manufacturer’s instructions. To amplify a fragment of the 16S rRNA gene that includes the V3 and V4 variable regions, primers 341F (5’-CCT ACG GGN GGC WGC AG-3’) and 805 R (5’-GAC TAC HVG GGT ATC TAA TCC-3’) (LC Sciences, Houston, TX) were used. The obtained amplicons were sequenced using the NovaSeq 6000, 2 × 250bp (NovaSeq 6000 SP Reagent Kit, 500 cycles). The resulting sequencing reads were analyzed using the bioinformatic pipeline Quantitative Insights Into Microbial Ecology (QIIME) 2 version 2022.2, with the Greengenes database v. 13_8 for taxonomic assignment to amplicon sequence variants (ASVs) clustered at a 97% similarity (cASV) using the Divisive Amplicon Denoising Algorithm (DADA2; 27214047).

### Data analysis and statistics

Statistical calculations, including partial least squares discriminant analysis (PLSDA), were carried out using R programming language (version 3.4.2). Differently abundant taxa between groups were identified using linear discriminant analysis (LDA) combined with effect size (LEfSe) algorithm^52;^ a cutoff value of LDA score (log10) above 2.0 was chosen. In addition, significantly different taxa were also identified by Mann-Whitney test on read abundances that underwent *centered log ratio* (CLR) transformation. Stratification based on catabotyping analysis was carried out based on the fecal concentrations of acetate, butyrate, propionate, lactate, succinate, and valerate using JSD distance and the Partitioning around Medoids (PAM) algorithm. Principal Coordinate Analysis with centroid was used to represent the samples graphically. For paired/unpaired matches with all the other variables considered in the study, paired/unpaired Student’s t-test or Mann-Whitney U and Wilcoxon Signed-Ranks tests were adopted depending on normal distribution assessed through the Shapiro-Francia test. Correlation analyses were carried out calculating Kendall’s τ rank correlation coefficient.

## Supplementary Material

Supplemental Material

## Data Availability

Metataxonomic raw sequencing data are available as FASTQ files in the European Nucleotide Archive (ENA) of the European Bioinformatics Institute under the accession code PRJEB64301. Processed data are included in the article or uploaded as supplementary materials. All the other data are available upon request.

## PROBE-IBS/2 group collaborators

Bruno Annibale, Roma; Guido Basilisco, Milano; Leonilde Bonfrate, Bari; Michele Cicala, Roma; Rocco Cosintino, Roma; Antonio Di Sabatino, Pavia; Raffaella Ferraro, Vercelli; Francesca Galeazzi, Padova; Bastianello Germanà, Belluno; Giovanni Maconi, Milano; Santino Marchi, Pisa; Gerardo Nardone, Napoli; Matteo Neri, Chieti; Fabio Pace, Seriate; Piero Portincasa, Bari; Franco Radaelli, Como; Marcello Rodi, Vercelli; Giovanni Sarnelli, Napoli; Paolo Usai, Cagliari.
