# Supplementary material for: Fecal short-chain fatty acids in non-constipated irritable bowel syndrome: a potential clinically relevant stratification factor based on catabotyping analysis
Source: Gut Microbes. 2023 Nov 1;15(2):2274128. doi: 10.1080/19490976.2023.2274128 (PMC10773536; doi:10.1080/19490976.2023.2274128)
Supplement: Supplemental Material [file KGMI_A_2274128_SM0458.docx]

**Supplementary Methods**

*Recommendations on Lifestyle and Dietary Habits*

The study incorporated specific guidelines for lifestyle and dietary practices. Participants were advised to avoid certain food items, including spicy food, soft drinks, coffee, and wine (small quantities were permitted only with meals). Consumption of milk and fresh milk products was restricted to minimal quantities, while long-ripened cheeses were allowed. Intake of spirits, fats (particularly when cooked), sausages with high fat content, red meat, game meat, fibrous and large-leaf vegetables (such as lettuce, red chicory, savoy cabbage, etc.), legumes, tomato preserves, underripe fruit with high fiber content (e.g., pineapple, citrus fruits), and whole foods (especially bran) was discouraged. Participants were encouraged to consume a substantial amount of vegetables with a high "soluble" fiber content, such as carrots, zucchini, and potatoes. Additionally, peeled and cooked fruit, such as apples and pears, was recommended. Regular physical exercise was emphasized, and participants were advised to avoid heavy meals and adhere to regular meal times. It was also suggested to chew food thoroughly and maintain adequate fluid intake, preferably in the form of still mineral water.

**Supplementary Figures**

**Figure S1**. Receiver Operator Curve (ROC) curve of the PLS discriminant analyses (PLSDA) shown in Figure 2.


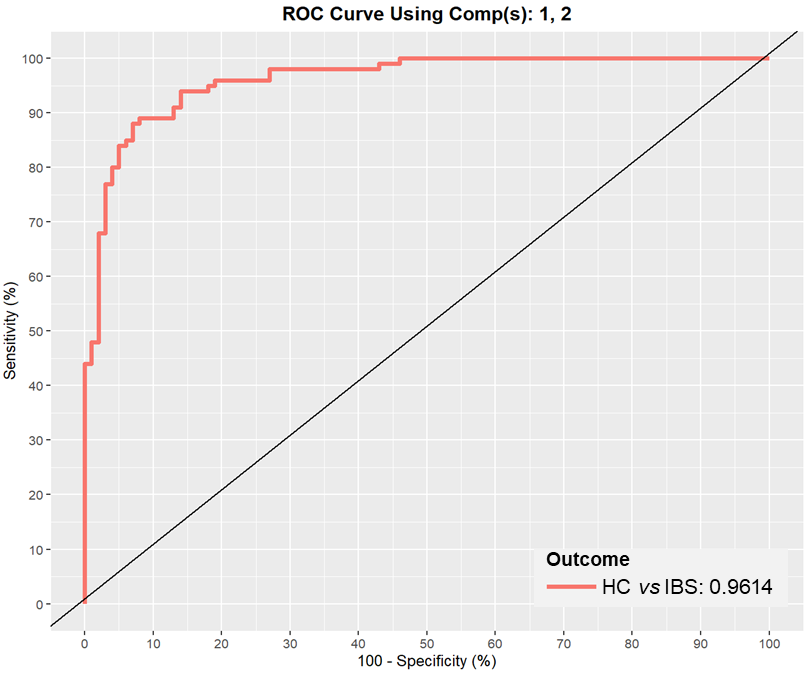


**Figure S2**. Analysis of the fecal microbiome of IBS-D patients, IBS-M patients, and healthy controls. **A**, intra-sample biodiversity according to four different α-diversity indexes. **B**, PLS discriminant analysis (PLSDA) with prediction background. ROC, Receiver Operating Characteristic of the PLSDA model. **C**, Concentration of organic acids in fecal samples reported in mmol/g of feces. Prp/But, ratio between the concentrations of propionate and butyrate. Statistics is according to Mann-Whitney test. *, P<0.05; **, P<0.01; ***, P<0.001; ****, P<0.0001.

**A**

**B**


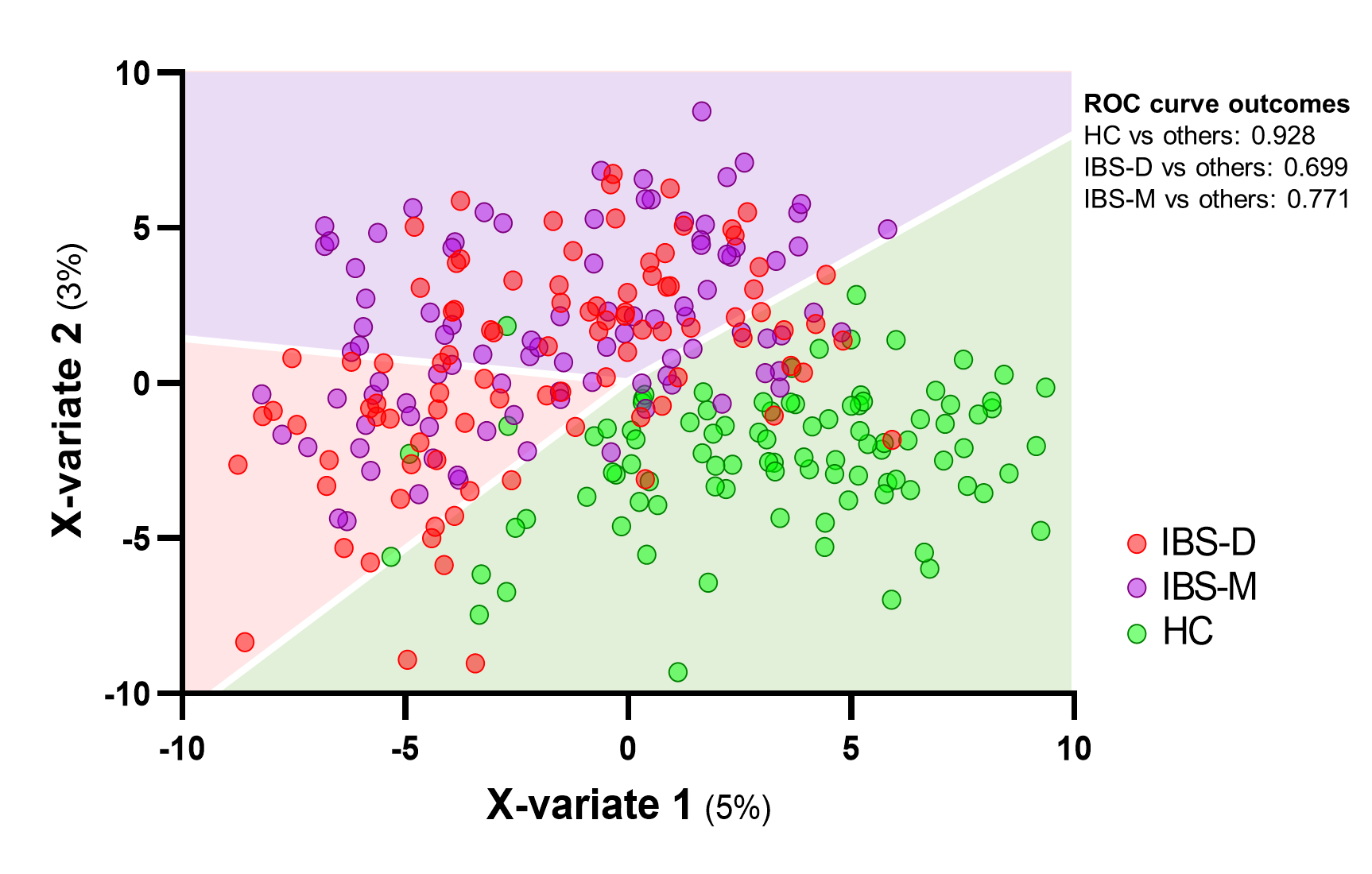


**C**

**
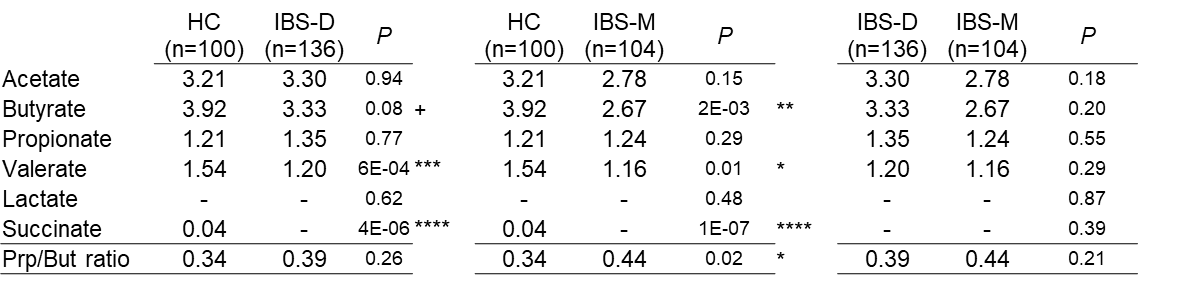
**

**Figure S3**. Histogram of the LDA scores (log10) computed for taxa with significantly differential abundance in IBS patients (NC-IBS) and healthy subjects (HC) deriving from the LEfSe analysis shown in Figure 4A. Positive LDA scores represent taxa significantly (p<0.05) higher in the NC-IBS group; negative LDA scores represent taxa significantly (p<0.05) higher in the HC group. The names of the taxonomic levels are abbreviated as follows: p, phylum; c, class; o, order; f, family; g, genus; s, species.


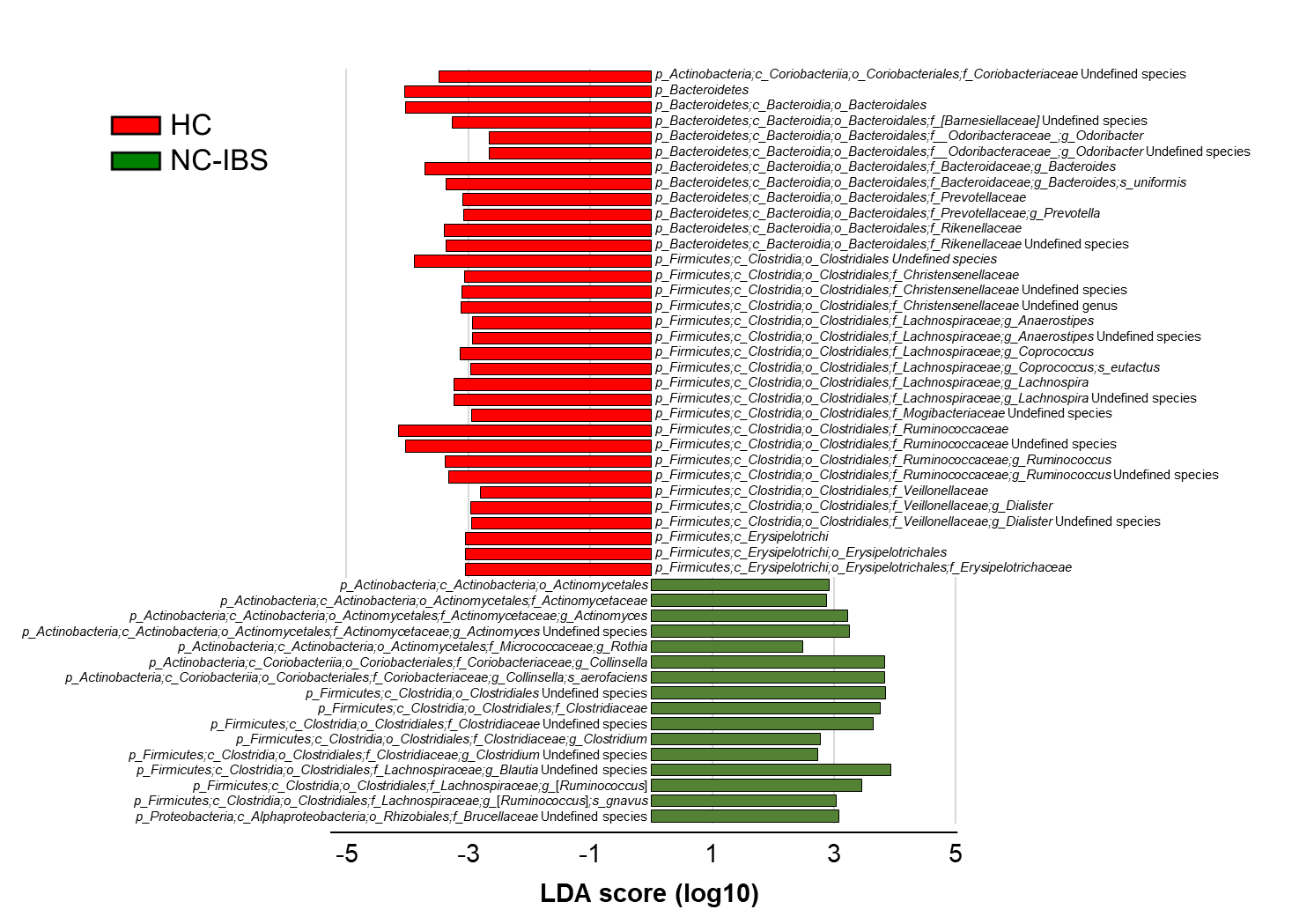


**Figure S4**. Comparison of the intra-sample biodiversity of the faecal bacterial communities between healthy controls (HC; n=100) and the two catabotypes of non-constipated IBS patients (FC-L, n=110; FC-H, n=138) according to four different α-diversity indexes. Statistics is according to Mann-Whitney test. **, P<0.01; ***, P<0.001; ****, P<0.0001).

**Figure S5**. Bacterial taxa in faecal samples exhibiting a significantly different abundance between healthy control (HC) and catabotype FC-L (**panel A**), and between HC and catabotype FC-L (**panel B**). Significantly different taxa have been determined through Mann-Whitney test carried out with CLR-transformed bacterial abundances. The black-yellow heatmap represents the mean CLR-transformed abundances of the reported taxonomic units. Higher and lower abundances for each taxon are reported with a red and cyan background, respectively. The taxonomic lineage of each taxon is shown: p, phylum; c, class; o, order; f, family; g, genus; s, species.

**A**

**B**
